# Relationship between volume and outcome for gastroschisis: a systematic review protocol

**DOI:** 10.1186/s13643-020-01462-y

**Published:** 2020-09-02

**Authors:** Johannes Morche, Tim Mathes, Anja Jacobs, Lucas Wessel, Edmund A. M. Neugebauer, Dawid Pieper

**Affiliations:** 1 Institute for Research in Operative Medicine, Faculty of Health, School of Medicine, Witten/Herdecke University, Ostmerheimer Str. 200, Building 38, 51109 Cologne, Germany; 2 Medical Consultancy Department, Federal Joint Committee, Gutenbergstraße 13, 10587 Berlin, Germany; 3 Department of Pediatric Surgery, Medical Faculty Mannheim (UMM), University of Heidelberg, Theodor-Kutzer-Ufer 1-3, 68167 Mannheim, Germany; 4 Brandenburg Medical School Theodor Fontane, Campus Neuruppin, Fehrbelliner Straße 38, 16816 Neuruppin, Germany

**Keywords:** Gastroschisis, Congenital anomalies, Hospitals, high-volume, Hospitals, low-volume, Hospital volume, Surgeon volume, Volume-outcome

## Abstract

**Background:**

Gastroschisis is a congenital anomaly that needs surgical management for repositioning intestines into the abdominal cavity and for abdominal closure. Higher hospital or surgeon volume has previously been found to be associated with better clinical outcomes for different especially high-risk, low volume procedures. Therefore, we aim to examine the relationship between hospital or surgeon volume and outcomes for gastroschisis.

**Methods:**

We will perform a systematic literature search from inception onwards in Medline, Embase, CENTRAL, CINAHL, and Biosis Previews without applying any limitations. In addition, we will search trial registries and relevant conference proceedings. We will include (cluster-) randomized controlled trials (RCTs) and prospective or retrospective cohort studies analyzing the relationship between hospital or surgeon volume and clinical outcomes. The primary outcomes will be survival and mortality. Secondary outcomes will be different measures of morbidity (e.g., severe gastrointestinal complications, gastrointestinal dysfunctions, and sepsis), quality of life, and length of stay. We will systematically assess risk of bias of included studies using RoB 2 for individually or cluster-randomized trials and ROBINS-I for cohort studies, and extract data on the study design, patient characteristics, case-mix adjustments, statistical methods, hospital and surgeon volume, and outcomes into standardized tables. Title and abstract screening, full text screening, critical appraisal, and data extraction of results will be conducted by two reviewers independently. Other data will be extracted by one reviewer and checked for accuracy by a second one. Any disagreements will be resolved by discussion. We will not pool results statistically as we expect included studies to be clinically and methodologically very diverse. We will conduct a systematic synthesis without meta-analysis and use GRADE for assessing the certainty of the evidence.

**Discussion:**

Given the lack of a comprehensive summary of findings on the relationship between hospital or surgeon volume and outcomes for gastroschisis, this systematic review will put things right. Results can be used to inform decision makers or clinicians and to adapt medical care.

**Systematic review registration:**

Open Science Framework (DOI: 10.17605/OSF.IO/EX34M; 10.17605/OSF.IO/HGPZ2)

## Background

Gastroschisis is a congenital anomaly with an incidence that increased during the last decades up to 2.7 to 4.9 cases per 10,000 live births based on current population studies [1–4]. It is characterized by a full-thickness defect in the abdominal wall, usually located to the right of the umbilical cord, which leads to extrusion of intestines or other organs into the amniotic fluid [5, 6]. The etiology is not fully understood so far, but maternal (e.g., young maternal age) and environmental teratogenic (e.g., smoking) factors seem to be of importance [7]. Gastroschisis can be categorized into simple and complex cases. Complex cases are associated with intestinal atresia, stenosis, perforation, or volvulus while simple cases are not associated with any of these intestinal pathologies. Therefore, complex cases have an increased mortality and morbidity compared to simple cases [8, 9]. Based on the results of a meta-analysis, intra-hospital mortality rate is about 17% for complex and 2% for simple gastroschisis. Results also show that risk of sepsis and short bowel syndrome is increased for complex cases and that length of parenteral nutrition as well as length of hospital stay is prolonged compared to simple cases [8].

Diagnosis of gastroschisis is usually made during prenatal ultrasound from the end of the first trimester [5]. Some fetal surgeons argue for amnioexchange in order to reduce digestive compounds in the amniotic fluid that are involved in inflammatory reactions. However, results of a recent randomized controlled trial indicate that amnioexchange should not be used for care in fetuses with simple gastroschisis [10]. Nevertheless, saline amnioinfusion might be beneficial for oligohydramnios in fetuses with gastroschisis [10–12].

There is no definitive evidence on the optimal mode (cesarean section vs. vaginal [13, 14]) and timing of delivery for neonates with gastroschisis [15, 16]. After delivery, initial treatment aims at maintaining the physiologic homeostasis with intravenous fluids, respiratory support if required, thermoregulation, and bowel protection [6]. Surgical management follows to reposition intestines into the abdominal cavity and to close the abdominal wall [17, 18]. It can be conducted with sutured fascial and/or skin closure (primary closure) or with placement of a silo followed by a delayed closure (staged closure) or by using the umbilical cord to cover the defect (sutureless closure) [17–19]. The staged closure is recommended if the neonate is unstable immediately after birth, if the reduction is likely to cause an abdominal compartment syndrome, or if herniated loops are very edematous, tightly matted together, and covered by a thick peel [6, 18]. Postoperative management includes adequate sedation and analgesia, parenteral nutrition, and mechanical ventilation if needed. The establishment of enteral nutrition depends on the initiation of gastrointestinal functions of the neonate. It is prolonged for complex cases compared to simple cases [6, 17].

Management of gastroschisis is not broadly standardized across institutions leading to variability in care between different centers [20]. However, different initiatives started to develop and introduce standardized protocols and pathways for the management and care of gastroschisis [21–23]. Systematic reviews on various other surgical procedures indicate a positive relationship between hospital as well as surgeon volume and clinical outcomes [24–27]. This relationship seems to be stronger for high-risk, low-volume procedures [28–31]. Given the characteristics of gastroschisis (long length of stay; need for hospital-based services; multidisciplinary care teams consisting of obstetricians, neonatologists, and pediatric surgeons) and the insights on the positive relationship between hospital volume and outcomes for other indications [29], it is plausible that such a relationship might also exist for gastroschisis. Taylor and Shew examined the effects of hospital and other health care system factors on outcomes [32]. In their non-systematic review, they conclude that “the majority of the evidence points to an improvement in gastroschisis outcomes when infants are born at, or treated at, higher volume centers and higher level NICUs” [32]. Regarding hospital volume, they refer to results of four primary studies [33–36]. Also, sound knowledge about the relationship between surgeon volume and outcomes for gastroschisis is important and can lead to methodological refinement of clinical studies on different surgical procedures. Without decent consideration of learning effects, trials might lead to better outcomes for established procedures only due to its longer existence and not due to the procedure itself [37]. Moreover, in general, only few multicenter trials report about provider effects due to variation in expertise which might cause misleading conclusions if low-volume and high-volume providers are included in the same trial [38].

To the best of our knowledge, there is no systematic review that analyzes the relationship between hospital or surgeon volume and outcomes for gastroschisis. Hence, it seems reasonable to conduct a systematic review on this issue. It is suggested that outcomes of neonates suffering from gastroschisis that are operated in a high volume hospital or by a high volume surgeon are favorable compared to outcomes of neonates that are operated in lower volume hospitals or by lower volume surgeons. The aim of our systematic review is to examine the available literature on the relationship between hospital as well as surgeon volume and outcomes for gastroschisis.

## Methods/design

This protocol is reported in accordance with the reporting guidance provided in the Preferred Reporting Items for Systematic Reviews and Meta-Analyses Protocols (PRISMA-P) statement (see checklist in additional file 1) [39]. The present protocol has been registered within the Open Science Framework platform (registration numbers 10.17605/OSF.IO/EX34M; 10.17605/OSF.IO/HGPZ2). Planned methods will be in line with those reported in our systematic review on the relationship between volume and outcomes for congenital diaphragmatic hernia [40, 41].

### Literature search strategy

We will perform a systematic literature search to identify all published studies on the relationship between hospital or surgeon volume and clinical outcomes for gastroschisis. Medline (via PubMed), Embase (via Ovid), CENTRAL (via Cochrane Library), CINAHL (via EBSCO), and Biosis Previews (via Ovid) will be searched from inception until the day of search. We will use a combination of controlled vocabulary terms (e.g., Medical Subject Headings (MeSH)) and free text words (a draft of the search strategy for Medline can be found in additional file 2). No language restrictions or other limits will be applied. Additionally, we will search trial registries (clinicaltrials.gov and the International Clinical Trials Registry Platform (ICTRP)). Reference lists of relevant articles will be inspected to identify additional articles that could have been missed by the search strategy. Additionally, we will screen individual conference proceedings (see additional file 3 for a list of conferences). Furthermore, we will use Google Scholar to identify grey literature. We will contact authors for detailed information in case of perceived relevance of abstracts. The search results will be uploaded and managed using Endnote.

### Eligibility criteria

The following inclusion criteria will be applied to each publication:

- Patients: We will include studies involving newborns with gastroschisis. We will not use a specific definition for gastroschisis but report the definition used in the corresponding studies.
- Exposure/control: Volume (i.e., hospital volume or surgeon volume) is the number of cases treated or surgeries conducted by a hospital or by a surgeon in a particular period of time. We will include studies if volume is assessed as a categorical variable or a continuous variable and if at least two different hospitals or surgeons are analyzed.
- Outcomes: We will include studies if at least one of the outcomes listed in the section “Outcomes and prioritization” is analyzed.
- Study design: We will include (cluster-) randomized controlled trials (RCTs) and prospective or retrospective cohort studies.
- Language: We will include studies written in English or German.

### Study selection

All titles and abstracts of articles identified through systematic literature search will be screened independently by two members of the research team. The full texts of potentially eligible articles will be obtained, and the eligibility of the full texts against the review inclusion criteria will be assessed by two reviewers independently. Any disagreements will be resolved by discussion. The study selection will be documented in Endnote.

### Data collection

For each included publication, the following characteristics will be extracted: year of publication, country, study design and methodology, data source, study period, definition of gastroschisis, number of patients, number of hospitals and/or surgeons, patient characteristics, case-mix adjustments, statistical methods, volume categories for hospitals, volume categories for surgeons, analyzed outcomes, results regarding these outcomes, and the funding source as well as authors’ reported conflicts of interest. With regards to wording, we use the term registry also for administrative databases. All data will be extracted into structured summary tables using Microsoft Word. We already piloted and used these tables for our systematic review on congenital diaphragmatic hernia [40, 41]. Results will be extracted independently by two reviewers. Other data will be extracted by one reviewer and checked for accuracy by a second reviewer who will read the paper in detail and ensure that no relevant information was missed. Any disagreements will be discussed until consensus is reached.

Study results will be recorded separately for each unit (surgeon or hospital) and outcome. If study authors present adjusted and unadjusted results, we will focus our synthesis on adjusted results. Nevertheless, we will extract unadjusted results as well. In case volume is classified in categories, we will report hazard ratios for time-to-event analyses and odds ratios or risk ratio for dichotomous outcomes and mean difference for continuous outcomes. We will provide the measures with 95% confidence levels if reported by authors or calculable given the available data. Moreover, we will recalculate effect measures (e.g., odds ratio, hazard ratio, risk ratio) so that higher volume is compared to lower volume and not vice versa. We will calculate effect measures if results can be calculated from information in the text but effect measures are not presented. In case volume is treated as a continuous variable, we will present results of the analyses conducted within primary studies. We will contact study authors for clarification in any case of uncertainty regarding data collection and critical appraisal.

### Outcomes and prioritization

The primary outcomes that will be analyzed in our systematic review are survival and mortality (surgery-related; up to discharge; long-term, e.g., 2-year or 5-year) given that gastroschisis is a life-threatening disease. Secondary outcomes are sepsis, growth (i.e., weight, length, and head circumference), number of operations, severe gastrointestinal complications (i.e., intestinal perforation; any intestinal resection, regardless of amount of bowel removed or the indication for the resection; mechanical intestinal obstruction resulting in a repeat laparotomy; abdominal compartment syndrome; enterocolitis), time on parenteral nutrition, liver disease (e.g., persistent conjugated hyperbilirubinemia (> 50 μmol/L) for ≥ 2 weeks with no known other underlying liver disease), and quality of life for the child as these outcomes have been included into a gastroschisis core outcome set in addition to death [42]. Moreover, we include length of stay, time on ventilation, amount of bowel resection in case of complex gastroschisis, gastrointestinal dysfunctions, and measures of neurodevelopment and cognition as they are also reported as being important outcomes [43–46].

### Risk of bias in individual studies

So far, there is no consensus on which tool to use for quality appraisal of studies on the relationship between volume and outcomes when conducting a systematic review [30]. These studies are almost exclusively based on observational data. We will use the tool for assessing risk of bias in non-randomized studies of interventions (ROBINS-I) that was recently developed by members of Cochrane methods groups [47] for assessing risk of bias of cohort studies. We already used ROBINS-I successfully when conducting our systematic review on the relationship between volume and outcomes for surgery on CDH [40, 41]. We will assess the risk of bias of adjusted results, if available. We will assess the effect of starting and adhering to intervention and use a cluster randomized trial as target trial. We will use the Cochrane risk-of-bias tool 2.0 (RoB 2) if any individually RCT will be identified [48]. We will use RoB 2 including special issues in assessing risk of bias in cluster-randomized trials mentioned in the Cochrane Handbook if any cluster-RCTs will be identified [49]. Methodological quality of the eligible studies will be assessed independently by two reviewers. Any disagreements will be resolved by discussion.

### Data synthesis

We expect the included studies to be clinically and methodologically diverse, e.g., including neonates with diverse illness severities (simple or complex gastroschisis) and applying different types of operative repair [17] as well as using different cut-off values for high and low volume [30, 31]. Therefore, we will provide a systematic synthesis without meta-analysis to summarize and explain the findings of the included studies. We will report our planned synthesis according to the Synthesis Without Meta-analysis (SWiM) guideline [50]. Due to the different prognosis for diverse illness severities [8, 9] and the different use of the types of operative repair [17–19], we will group studies according to illness severity (simple or complex gastroschisis) and types of operative repair (primary closure, staged closure, sutureless closure), if possible. We will present findings of included studies in tables, and we will structure studies in the tables by risk of bias of the systematic review’s primary outcomes and study size in case of a similar risk of bias. Also, we will use these criteria to prioritize results for summary and synthesis. We will focus our synthesis on adjusted results and consider point estimates and 95% confidence intervals for conclusions. We will investigate whether results for diverse illness severities and types of different repair differ if possible. The certainty of the evidence will be assessed by using GRADE. We will synthesize findings based on outcomes separately for each unit (surgeon or hospital) and consider risk of bias, imprecision, indirectness, inconsistency, and publication bias as well as the magnitude of treatment (or exposure) effect, the presence of a dose–response gradient, and plausible residual confounding as recommended by GRADE [51, 52]. We are aware that the risk of publication bias is particularly high for studies based on automatically collected observational data (e.g., in electronic medical records or registries) [53]. However, to our knowledge, there is no tool to assess presence and extent of publication bias when results are not pooled across studies. Therefore, we will discuss potential impact of publication bias narratively.

The proposed systematic review will be reported in accordance with the reporting guidance provided in the Preferred Reporting Items for Systematic Reviews and Meta-analyses (PRISMA) statement [54]. Any amendments made to this protocol when conducting the study will be outlined and reported in the final manuscript.

## Discussion

The aim of this review is to evaluate the relationship between hospital or surgeon volume and outcomes for gastroschisis. So far, there is no up-to-date analysis that synthesizes results from different studies on the volume-outcome relationship systematically and comprehensively. Therefore, it is important to evaluate this relationship so that insights can be used to inform decision makers or clinicians and to adapt medical care.

However, based on previous research, we do not expect to find any relevant (cluster-)RCT so that our systematic review will rely on findings from cohort studies. This might limit the certainty of our conclusions. As we restrict eligible studies to English and German documents, this might introduce language bias. Also, based on experience from previous work, we expect it sometimes to be difficult to assess whether volume in the primary studies refers to the number of cases treated or to the number of surgeries performed by a hospital or by a surgeon. We plan to disseminate results of our systematic review through publication in a peer-reviewed journal.

## Supplementary information

**Additional file 1:.** PRISMA-P 2015 Checklist

**Additional file 2:.** List of conferences

**Additional file 3:.** Search strategy for Medline (Pubmed)

## Data Availability

Not applicable

## Abbreviations

CENTRAL: Cochrane Central Register of Controlled Trials
CINAHL: Cumulative Index to Nursing and Allied Health Literature
GRADE: The Grading of Recommendations Assessment, Development and Evaluation
RCT: Randomized controlled trial
ROBINS-I: Risk of bias in non-randomized studies of intervention
