# Supplementary material for: Relationship between volume and outcome for gastroschisis: a systematic review protocol
Source: Syst Rev. 2020 Sep 2;9:203. doi: 10.1186/s13643-020-01462-y (PMC7469094; doi:10.1186/s13643-020-01462-y)
Supplement: Supplementary file 2 — Additional file 2:. List of conferences [file 13643_2020_1462_MOESM2_ESM.docx]

**Additional file 2: Search strategy for Medline (Pubmed)**

Medline (Pubmed)

| # | #Suchfrage |
| --- | --- |
| #1 | Gastroschisis[mh] |
| #2 | Gastroschis*[tiab] |
| #3 | Laparoschis*[tiab] |
| #4 | “congenital fissure”[tiab] |
| #5 | “congenital fissures”[tiab] |
| #6 | Abdominal wall[mh] |
| #7 | “abdominal wall defect”[tiab] |
| #8 | “abdominal wall defects”[tiab] |
| #9 | #1 OR #2 OR #3 OR #4 OR #5 OR #6 OR #7 OR #8 |
| #10 | "hospitals, low-volume"[mh] |
| #11 | "hospitals, high-volume“[mh] |
| #12 | "workload"[mh] |
| #13 | ("hospitals/statistics and numerical data"[mh]) |
| #14 | ("hospitals, pediatric/statistics and numerical data"[mh]) |
| #15 | ("intensive care units, pediatric/statistics and numerical data"[mh]) |
| #16 | ("intensive care, neonatal/statistics and numerical data"[mh]) |
| #17 | volume*[tiab] |
| #18 | regionali*[tiab] |
| #19 | centrali*[tiab] |
| #20 | workload*[tiab] |
| #21 | caseload*[tiab] |
| #22 | centre*[tiab] |
| #23 | center*[tiab] |
| #24 | size*[tiab] |
| #25 | experience*[tiab] |
| #26 | #10 OR #11 OR #12 OR #13 OR #14 OR #15 OR #16 OR #17 OR #18 OR #19 OR #20 OR #21 OR #22 OR #23 OR #24 OR #25 |
| #27 | "Outcome Assessment, Health Care"[mh] |
| #28 | outcom*[tiab] |
| #29 | Mortality[mh] |
| #30 | mortalit*[tiab] |
| #31 | surviv*[tiab] |
| #32 | „length of stay“[mh] |
| #33 | Sepsis[mh] |
| #34 | Growth[mh] |
| #35 | Reoperation[mh] |
| #36 | “Postoperative complications”[mh] |
| #37 | “intestinal diseases”[mh] |
| #38 | (intestinal[tiab]) AND (((perforation*[tiab]) OR obstruction*[tiab]) OR resection*[tiab]) |
| #39 | “Compartment Syndromes”[mh] |
| #40 | “Compartment syndrome”[tiab] |
| #41 | “parenteral nutrition”[mh] |
| #42 | “liver diseases”[mh] |
| #43 | “liver disease”[tiab] |
| #44 | Bilirubin[tiab] |
| #45 | “quality of life”[mh] |
| #46 | “Respiration, Artificial”[mh] |
| #47 | “neurodevelopmental disorders”[mh] |
| #48 | #27 OR #28 OR #29 OR #30 OR #31 OR #32 OR #33 OR #34 OR #35 OR #36 OR #37 OR #38 OR #39 OR #40 OR #41 OR #42 OR #43 OR #44 OR #45 OR #46 OR #47 |
| #49 | #9 AND #26 AND #48 |
