# Supplementary material for: Relationship between volume and outcome for gastroschisis: a systematic review protocol
Source: Syst Rev. 2020 Sep 2;9:203. doi: 10.1186/s13643-020-01462-y (PMC7469094; doi:10.1186/s13643-020-01462-y)
Supplement: Supplementary file 3 — Additional file 3:. Search strategy for Medline (Pubmed) [file 13643_2020_1462_MOESM3_ESM.docx]

**Additional file 3: List of conferences**

List of conferences:

Annual Meeting of the Canadian Association of Pediatric Surgeons

American Congress on Pediatric Surgery (APSA)

British Congress of Pediatric Surgery (BAPS)

European Congress of Pediatric Surgery (EUPSA)

International Conference on Maternal Fetal Neonatal Medicine

International Conference on Pediatrics and Pediatric Surgery (EUPSA, WOFAPS)

Jahrestagung der Deutschen Gesellschaft für Kinderchirurgie
